# HIV Testing and HIV Serostatus-Specific Sexual Risk Behaviour Among Men Who Have Sex with Men Living in England and Recruited Through the Internet in 2001 and 2008

**DOI:** 10.1007/s13178-012-0106-1

**Published:** 2012-12-02

**Authors:** Ford Hickson, Chris Bonell, James Hargreaves, David Reid, Peter Weatherburn

**Affiliations:** 1 Sigma Research, Department of Social and Environmental Health Research, Faculty of Public Health & Policy, London School of Hygiene and Tropical Medicine, 15-17 Tavistock Place, London, WC1H 9SH UK; 2 Department of Social Policy and Intervention, University of Oxford, Oxford, UK; 3 Department of Infectious Disease Epidemiology, London School of Hygiene and Tropical Medicine, London, UK

**Keywords:** Homosexuality, Male, Anal sex, Risk reduction, HIV-1, England

## Abstract

Using data from two large internet-recruited surveys in England in 2001 and 2008, we examine HIV status-specific patterns of unprotected anal intercourse (UAI). In adjusted comparisons between our 2008 and 2001 samples, there was evidence of a greater proportion of men living with diagnosed HIV, a reduction in sexual partners and in UAI with partners of unknown HIV status among men not tested HIV positive, increases in anal intercourse and UAI among men with diagnosed HIV and an increase in insertive UAI with HIV-positive men among men never tested for HIV. However, we found no evidence for increases in negotiated safety or sero-sorting. The data are compatible with a concentration of sexual risk among men with diagnosed HIV, countering an overall trend towards less risk taking among men not tested HIV positive.

## Introduction

Sex between men remains the most common context in which HIV is transmitted in the UK (Health Protection Agency 2011) and unprotected anal intercourse (UAI) remains the primary risk behaviour (Macdonald et al. 2008). Despite constantly rising HIV diagnoses among men-who-have-sex-with-men (MSM) throughout the 2000s, overall levels of UAI appear not to have changed during the same period. For example, Knussen et al. (2011) observed an increase towards the end of the 1990s in the proportion of MSM (recruited in gay venues in Glasgow and Edinburgh) who had multiple UAI partners but found no evidence for change in this risk behaviour between 2002 and 2008. Similarly, among MSM recruited in London gyms, Lattimore et al. (2011) observed an increase in the proportion reporting UAI between 1998 and 2001, but no change between 2001 and 2008. However, within these stable aggregate levels of UAI, there may be changes in the context within which UAI occurs, particularly in men’s knowledge of their own HIV status and that of their sexual partner, as well as in the modality of UAI engaged in (i.e. insertive or receptive).

Improvements in HIV treatments since the late 1990s have led to increased longevity for many of those diagnosed with HIV. In association with continuing new infections, this has resulted in an increase in the number of MSM living with diagnosed HIV (Health Protection Agency 2010). At the same time, increases in testing (Grulich and Kaldor 2008) are likely to have led to more HIV-negative men knowing their HIV status. The majority of HIV tests in the UK happen at genito-urinary medicine clinics where, in the early 2000s, HIV testing policy changed from opt-in to opt-out testing, greatly increasing the number of tests taken. Between 2001 and 2008, the number of men living in England with diagnosed homosexually acquired HIV and in touch with services almost doubled from 12,312 to 23,597 (Brian Rice, Health Protection Agency, personal communication, 18 August 2010).

Along with increases in HIV testing, since the mid-1990s, various forms of HIV status-specific risk reduction strategies have been reported (Fengyi et al. 2009). Men who have recently tested are better able to choose whether or not to engage in specific sexual acts on the basis of knowledge of their HIV concordancy with their sexual partner. Risk reduction tactics described among MSM include negotiated safety, sero-sorting, strategic positioning and viral load monitoring.

An increase in the proportion of men who are confident they are in HIV-negative-concordant relationships may result in an increase in unprotected intercourse within such relationships, which in conjunction with explicit rules about avoiding unprotected intercourse with other partners has been termed ‘negotiated safety’ (Kippax et al. 1993). If the increase in testing has resulted in an increase in negotiated safety agreements in relationships, we might expect an increase in UAI with HIV-negative partners among men tested HIV negative, a trend observed by Lattimore et al. (2011). ‘Sero-sorting’ refers to the more general practice of attempting to limit UAI to regular or casual partners of the same HIV status (Anonymous 2004)—a term sometimes limited to men with diagnosed HIV infection. If sero-sorting has become more common, we might expect a decline in men engaging in UAI with partners of sero-discordant or unknown status and/or an increase in UAI with partners thought to be concordant, both of which were reported by Lattimore et al. (2011). ‘Strategic positioning’ refers to the practice of attempting to avoid UAI where an HIV-positive man is insertive with an HIV-negative partner but not avoiding the reverse, since the former modality has a higher probability of transmission than the latter (Van de Ven et al. 2002). If strategic positioning was more commonly adopted over time, we might expect a reduction in insertive UAI with negative and unknown-status partners among men diagnosed with HIV and/or a reduction in receptive UAI with positive and unknown-status partners among men not tested HIV positive. Previous studies have not examined mode of UAI.

In this paper, we describe patterns of HIV testing and sexual risk behaviour in 2001 and in 2008 among men recruited over the internet to a community-based survey and explore evidence from these surveys of changes over time.

## Methods

The Gay Men’s Sex Survey was an annual, community-based, sexual health needs assessment survey for MSM in England that occurred each summer between 1997 and 2008. In both 2001 and 2008, data were collected on HIV testing and mode-specific sexual risk behaviours using identical questions with 12-month recall periods.

The surveys were built and administered using Demographix (Demographix Limited, London) online software and imported to SPSS Statistics 19 (IBM, New York). The opening page detailed the research aims and objectives, explaining participation was voluntary and giving a contact number should they have further questions. Completion was taken as indicating informed consent. Refusals could not be recorded so we cannot report response rates. While data from incomplete surveys were not collected, records of page views allow us to estimate that in 2008, 62 % of men who opened the first page of the survey completed it and submitted their answers. This information was not available for 2001. The surveys were approved by Portsmouth University Research Ethics Committee.

The two surveys originally used three different recruitment methods: paper questionnaires for on-the-spot completion at community events (2001 only); booklet questionnaires distributed by community organisations for return by post (both years); and internet-based questionnaires advertised on a range of websites. For the present analysis, we have excluded men recruited at community events (as this occurred in 2001 only) and those recruited through the booklet as these were distributed by a different array of health promotion and community organisations in the 2 years.

Online participants were recruited through internet banner advertisements on dating sites, news sites and community health promotion organisation sites. In 2001, a total of 3,682 men (living in England, aged 16 years and over and who had sex with a man in the last 12 months) were recruited through three websites (Gay.com, Sigma Research and one health promotion provider organisation), 96.3 % through the first named source. By 2008, Gay.com (a dating and news site) has closed and so was unavailable for recruitment. Instead, a total of 3,486 men (with the same inclusion criteria) were recruited through 38 different web sites. While the majority of these sites were health promotion providers and non-commercial gay community organisations, two sites together provided the same services as Gay.com had, namely Gaydar (a dating site) and Pink News (a news site).

A comparison of the Gaydar/Pink News recruits with all other recruits in 2008 suggested the latter had on average fewer sexual partners and were drawn from a less sexually active population. The Gaydar/Pink News recruits on the other hand had a comparable number of sexual partners as the Gay.com recruits in 2001. In order to make the recruitment sites as comparable as possible across the 2 years, we therefore selected for comparison respondents recruited through Gay.com in 2001 and through Gaydar or Pink News in 2008. This resulted in 3,517 men in the 2001 survey (recruited through Gay.com) and 1,382 men in the 2008 survey (1,082 recruited through Gaydar and 300 through Pink News).

Inclusion criteria for this paper were: aged 16 (the age of sexual consent in England) or over; living in England; and had sex with a man in the last year. In addition, men were asked to indicate their highest educational qualification and ethnic group. Men were allocated to one of three groups on the basis of their highest educational qualification. Those with no qualifications or GCE, CSE or GCSE certificates were classified as having ‘low’ educational qualifications. Those who indicated a degree or greater were classified as having ‘high’ educational qualifications. The remainder were classified as having ‘medium’ educational qualifications, including all those with A levels or equivalent and the majority of those with vocational or trade qualifications.

Behaviours of interest included HIV testing, any engagement in anal intercourse (AI), broken down by whether receptive or insertive, whether unprotected by condoms and with partners of known HIV-positive, HIV-negative and/or unknown HIV statuses. The proportions engaging in each behaviour in each year are presented.

Although changes occurred to the way in which men were recruited between 2001 and 2008, we recognise that it is of particular interest to explore evidence for changes in practice that had occurred over time. We therefore present odds ratios for engagement in HIV testing and risk behaviours comparing men recruited in 2008 with those recruited in 2001. We used logistic regression to calculate crude measures of effect and then adjusted these for potential socio-demographic confounders. Through this method, we hoped to adjust for sampling differences between the surveys and to cautiously interpret our findings in relation to hypothesised underlying changes over time rather than differences between the samples.

## Results

### Demographic Differences Between the Two Survey Samples

Table 1 shows the region of residence, age, ethnicity and education level of the two samples. There were statistically significant differences in all four characteristics across the 2 years. Relative to 2001, the 2008 survey recruited slightly larger proportions living in London, West Midlands and the South West and fewer men in all other regions except the South East Coast which remained unchanged.

**Table 1:** Characteristics of two internet-recruited samples of MSM in England, 2001 and 2008

		Number of respondents (%)		*p* value
		2001, *N =* 3,517 (%)	2008, *N* = 1,382 (%)	
Region of residence	London	1,031 (29.3)	437 (31.6)	0.006
	South West	273 (7.8)	142 (10.3)	
	South Central	279 (7.9)	97 (7.0)	
	South East Coast	303 (8.6)	119 (8.6)	
	East of England	291 (8.3)	109 (7.9)	
	East Midlands	232 (6.6)	84 (6.1)	
	West Midlands	257 (7.3)	122 (8.8)	
	Yorkshire and Humber	265 (7.5)	79 (5.7)	
	North West	469 (13.3)	155 (11.2)	
	North East	117 (3.3)	38 (2.7)	
Age (years)	<20	409 (11.6)	56 (4.1)	<0.001
	20–29	1,407 (40.0)	396 (28.7)	
	30–39	1,036 (29.5)	387 (28.0)	
	40–49	480 (13.6)	348 (25.2)	
	50+	185 (5.3)	195 (14.1)	
Ethnic group	White British	2,949 (83.9)	1,109 (80.4)	0.034
	White other	360 (10.2)	165 (12.0)	
	Asian and Asian–White	83 (2.4)	39 (2.8)	
	Black and Black–White	52 (1.5)	31 (2.2)	
	All others	69 (2.0)	36 (2.6)	
Education level	Low	885 (25.2)	289 (20.9)	<0.001
	Medium	1,140 (32.4)	327 (23.7)	
	High	1,476 (42.0)	764 (55.4)	

There were missing data on age in 2001 (*n* = 3), ethnic group in 2001 (*n* = 3) and 2008 (*n* = 2) and education level in 2001 (*n* = 16) and 2008 (*n* = 2). Those with missing data were excluded from further multivariate analyses

The 2008 sample (median age 36 years, range 16–83, mean 36.6, standard deviation 11.86) was significantly older than the 2001 sample (median age 29 years, range 16–73, mean 30.7, standard deviation 10.00: *F* = 313.69, *df* = 1, *p* < .001), containing as it did a smaller proportion of men under 30 and a higher proportion of men aged 40 and older. This may reflect the early uptake of the internet by younger men and its gradual adoption by older men over the period under consideration.

Relative to the 2001 sample, the 2008 sample had fewer White British men and more men in each of the ethnic minority groups. The 2008 sample was more highly educated than the 2001 sample, with fewer men having low or medium education and more having high education.

### HIV Testing History

Whether or not men had ever reported testing for HIV and what their last reported test result was is shown in Table 2 for the 2 years. The proportion of men who reported ever testing for HIV was 45.8 % in 2001 and 69.3 % in 2008. The proportion living with diagnosed HIV was 3.4 % in 2001 and 9.1 % in 2008. The proportion reporting never having tested was 54.2 % in 2001 and 30.7 % in 2008.

**Table 2:** HIV testing history among MSM recruited over the internet in England in 2001 and 2008

HIV test history	2001, *N* = 3,499 (%)	2008, *N* = 1,378 (%)	OR (95 % CI) comparing 2008 vs 2001^a^	
			Crude	Adjusted^a^
Never	1,897 (54.2)	423 (30.7)	0.37 (0.33–0.43)	0.45 (0.39–0.51)
Negative	1,484 (42.4)	830 (60.2)	2.06 (1.81–2.34)	1.78 (1.56–2.04)
Positive	118 (3.4)	125 (9.1)	2.85 (2.20–3.71)	2.31 (1.76–3.03)

^a^Adjusted for residence, age, ethnic group and education

Adjusting for differences in residence, age, ethnic group and education, men participating in the survey in 2008 were significantly less likely to report never having had an HIV test than those participating in 2001. Both the proportion reporting living with diagnosed HIV and the proportion reporting a previous negative test result had increased over time.

### Aggregate Sexual Risk Behaviours

In both years, slightly over half of respondents reported five or more male sexual partners in the preceding 12 months (Table 3). Any engagement in anal intercourse in the 12-month period was very commonly reported in both years.

**Table 3:** Sexual risk behaviours among MSM recruited over the internet in England in 2001 and 2008

Measure (in last 12 months)		2001, *n*/*N* (%)	2008, *n*/*N* (%)	OR (95 % CI) comparing 2008 vs. 2001	
				Crude	Adjusted^c^
Male sex partners	1–4	1,647/3,497 (47.1)	639/1,382 (46.2)	0.97 (0.85–1.10)	1.07 (0.94–1.21)
	5+	1,850/3,497 (52.9)	743/1,382 (53.8)	1.04 (0.91–1.17)	0.94 (0.82–1.07)
Any AI		3,065/3,435 (89.2)	1,223/1,355 (90.3)	1.12 (0.91–1.37)	1.26 (1.02–1.59)
Any UAI		2,025/3,435 (59.0)	779/1,355 (57.5)	0.94 (0.83–1.07)	1.08 (0.95–1.24)
Any UAI-pos		127/3,385 (3.8)	101/1,368 (7.4)^b^	2.05 (1.56–2.68)	1.80 (1.36–2.38)
Any UAI-neg		1,005/3,399 (29.6)	400/1,371 (29.2)	0.98 (0.86–1.13)	1.11 (0.96–1.28)
Any UAI-dk		1,214/3,407 (35.6)	420/1,368 (30.7)^b^	0.80 (0.70–0.92)	0.88 (0.77–1.01)
Any RAI		2,556/3,494 (73.2)	984/1,377 (71.5)	0.92 (0.80–1.06)	1.06 (0.91–1.22)
Any RUAI		1,577/3,482 (45.3)	580/1,377 (42.1)^a^	0.88 (0.78–0.99)	1.03 (0.90–1.18)
Any IAI		2,663/3,486 (76.4)	1,004/1,382 (72.6)^b^	0.82 (0.71–0.95)	0.89 (0.77–1.03)
Any IUAI		1,594/3,466 (46.0)	577/1,381 (41.8)^b^	0.84 (0.74–0.95)	0.94 (0.82–1.07)

*AI* anal intercourse, *PAI* protected (with condom) AI, *UAI* unprotected (without condom) AI, *UAI-pos* UAI with a known HIV positive partner, *UAI-neg* UAI with a known HIV-negative partner, *UAI-dk* UAI with a partner of unknown HIV status, *RAI* receptive AI, *RUAI* unprotected (without condom) RAI, *IAI* insertive AI, *IUAI* unprotected (without condom) IAI

^a^Chi-squared significant over the two samples, *p* < 0.05

^b^Chi-squared significant over the two samples, *p* < 0.01

^c^Adjusted for area of residence, age, ethnic group and education

In our adjusted analysis of differences over time, we found no overall change in the reported number of sexual partners between the two surveys. We found a slight but significant increase in the proportion of men reporting engaging in anal intercourse in the 12-month period. However, there was little evidence for a change in the proportions reporting engaging in any unprotected anal intercourse (either receptive or insertive) or in the proportions reporting doing so with partners thought HIV negative or partners of unknown HIV status. There was a significant increase in the small proportion who reported engaging in UAI with a partner thought to be HIV positive.

### Sexual Risk Behaviour and HIV Status Concordancy Knowledge

Table 4 shows the same measures of sexual risk behaviour for the three testing history groups separately in both years. In addition, it shows the proportion reporting each of the mode-specific behaviours with partners thought to be HIV positive, HIV negative and whose HIV status was unknown.

**Table 4:** HIV sero-specific sexual risk behaviours among MSM recruited over the internet in England in 2001 and 2008, by HIV testing history

Measure (in last 12 months)		Men never tested for HIV			Men last tested HIV negative			Men diagnosed HIV positive		
		2001, *n*/*N* (%)	2008, *n*/*N* (%)	OR adjusted (95 % CI)^c^	2001, *N* (%)	2008, *N* (%)	OR adjusted (95 % CI)^c^	2001, *N* (%)	2008, *N* (%)	OR adjusted (95 % CI)^C^
Male sex partners	1–4	1,045/1,881 (55.6)	265/423 (62.6)^b^	1.44 (1.15–1.80)	555/1,480 (37.5)	343/830 (41.3)^a^	1.20 (1.00–1.43)	39/118 (33.1)	28/125 (22.4)	0.63 (0.35–1.14)
	5+	836/1,881 (44.4)	158 (37.4)^b^	0.69 (0.56–0.87)	925/1,480 (62.5)	487/830 (58.7)^a^	0.84 (0.70–0.99)	79/118 (66.9)	97/125 (77.6)	1.59 (0.88–2.90)
Any AI		1,572/1,836 (85.6)	350/415 (84.3)	1.04 (0.76–1.41)	1,367/1,465 (93.3)	750/813 (92.3)	1.01 (0.72–1.43)	112/118 (94.9)	121/123 (98.4)	11.1 (1.39–100.0)
Any UAI		990/1,836 (53.9)	195/415 (47.0)^a^	0.88 (0.71–1.10)	938/1,465 (64.0)	482/813 (59.3)	0.94 (0.78–1.13)	87/118 (73.7)	101/123 (82.1)	2.13 (1.09–4.17)
Any UAI-pos		13/1,804 (0.7)	8/418 (1.9)^a^	2.42 (0.97–6.05)	55/1,448 (3.8)	29/823 (3.5)	0.90 (0.56–1.43)	58/117 (49.6)	64/123 (52.0)	1.32 (0.77–2.27)
Any UAI-neg		414/1,810 (22.9)	90/419 (21.5)	1.05 (0.81–1.37)	563/1,456 (38.7)	289/825 (34.9)	0.96 (0.80–1.16)	23/117 (19.7)	22/123 (17.9)	1.04 (0.52-2.05)
Any UAI-dk		634/1,820 (34.8)	104/418 (24.9)^b^	0.68 (0.53–0.87)	521/1,453 (35.9)	254/569 (30.9)^a^	0.88 (0.73–1.06)	53/118 (44.9)	61/123 (49.6)	1.30 (0.76–2.22)
Any RAI		1,297/1,878 (69.1)	270/422 (64.0)^a^	0.94 (0.74–1.18)	1,146/1,480 (77.4)	604/826 (73.1)^a^	0.90 (0.73–1.10)	102/118 (86.4)	108/125 (86.4)	1.41 (0.63–3.14)
Any RUAI		754/1,870 (40.3)	145/42 (34.4)^a^	0.92 (0.73–1.15)	737/1,477 (49.4)	348/826 (42.1)^b^	0.86 (0.72–1.02)	78/118 (66.1)	86/125 (68.8)	1.43 (0.80–2.56)
Any RUAI-pos		6/1,846 (0.3)	2/421 (0.5)	1.42 (0.27–7.41)	28/1,473 (1.9)	14/826 (1.7)	0.98 (0.50–1.91)	47/118 (39.8)	51/123 (41.5)	1.25 (0.72–2.16)
Any RUAI-neg		330/1,846 (17.9)	67/421 (15.9)	1.00 (0.74–1.34)	454/1,473 (30.8)	210/826 (25.4)^b^	0.88 (0.72–1.07)	20/118 (16.9)	20/123 (16.3)	1.17 (0.58–2.40)
Any RUAI-dk		438/1,846 (23.7)	78/421 (18.5)^a^	0.83 (0.63–1.09)	370/1,473 (25.1)	167/826 (20.2)^b^	0.86 (0.70–1.07)	47/118 (39.8)	52/123 (42.3)	1.25 (0.73–2.16)
Any IAI		1,350/1,871 (72.2)	269/423 (63.6)^b^	0.76 (0.60–0.95)	1,200/1,479 (81.1)	632/830 (76.1)^b^	0.81 (0.66–1.01)	98/118 (83.1)	102/125 (81.6)	1.08 (0.53–2.20)
Any IUAI		770/1,857 (41.5)	130/423 (30.7)^b^	0.72 (0.57–0.90)	744/1,476 (50.4)	373/829 (45.0)^a^	0.90 (0.75–1.07)	73/117 (62.4)	74/125 (59.2)	0.98 (0.57–1.70)
Any IUAI-pos		9/1,832 (0.5)	8/420 (1.9)	3.67 (1.37–9.86)	45/1,455 (3.1)	25/827 (3.0)	0.94 (0.56–1.56)	51/117 (43.6)	48/125 (38.4)	0.84 (0.49–1.44)
Any IUAI-neg		307/1,832 (16.8)	59/420 (14.0)	0.93 (0.68–1.26)	409/1,455 (28.1)	209/827 (25.3)	0.97 (0.79–1.19)	17/117 (14.5)	12/125 (9.6)	0.76 (0.33–1.75)
Any IUAI-dk		479/1,832 (26.1)	65/420 (15.5)^b^	0.57 (0.42–0.76)	386/1,455 (26.5)	189/827 (22.9)	0.88 (0.72–1.08)	38/117 (32.5)	42/125 (33.6)	1.20 (0.68–2.11)

*AI* anal intercourse, *PAI* protected (with condom) AI, *UAI* unprotected (without condom) AI, *UAI-pos* UAI with a known HIV-positive partner, *UAI-neg* UAI with a known HIV-negative partner, *UAI-dk* UAI with a partner of unknown HIV status, *RAI* receptive AI, *RUAI* unprotected (without condom) RAI, *RUAI*-*pos* RUAI with a known HIV-positive partner, *RUAI-neg* RUAI with a known HIV-negative partner, *RUAI-dk* RUAI with a partner of unknown HIV status, *IAI* insertive AI, *IUAI* unprotected without condom) IAI, *IUAI-pos* IUAI with a known HIV-positive partner, *IUAI-neg* IUAI with a known HIV-negative partner, *IUAI-dk* IUAI with a partner of unknown HIV status

^a^Chi-squared significant over the two samples, *p* < 0.05

^b^Chi-squared significant over the two samples, *p* < 0.01

^c^Comparing 2008 vs. 2001 for behaviour measures

^d^Adjusted for area of residence, age, ethnic group and education

In adjusted analysis, there was some evidence that differences between the two surveys differed by HIV testing history. Although we observed no overall difference between the 2 years in the numbers of reported sexual partners, among men reporting not being tested HIV positive (those either never tested or tested negative) reported partner numbers were lower in 2008 than in 2001. Among men reporting testing HIV positive, the opposite direction of change was observed between the two surveys although this was not statistically significant.

Among men with diagnosed HIV, more men reported AI in 2008 than 2001. While very common in 2001, this was almost universal in 2008 (resulting in large confidence interval for the odds ratio). Reporting any UAI was significantly more common in 2008 than 2001 among men reporting being HIV positive while the change over time was downward in the other two groups. Reports of UAI with partners of unknown HIV status were less common in 2008 than 2001 among men reporting never having tested and among those reporting having tested negative, but not among men reporting having been diagnosed with HIV.

Among men reporting never having tested for HIV, a lower proportion reported UAI with a partner of unknown HIV status in 2008 than in 2001 in both modalities, but the adjusted odds ratio was significant only for insertive intercourse. There were significantly more men in 2008 than in 2001 reporting insertive UAI with partners thought to be HIV positive among this group. After adjusting for demographic differences in the samples, there were no reported modality-specific changes among either men reporting being diagnosed with HIV or those who reported testing HIV negative, either overall or with partners of specific HIV statuses.

## Discussion

### Summary of Main Findings

In our two large internet-based surveys of men who have sex with men recruited over the internet, we recruited more men who reported living with diagnosed HIV and considerably fewer men never having tested for HIV in 2008 than in 2001. However, in 2008, in our sample, almost a third of men reported they had not tested. Prior literature suggested that we might expect a reduction over time in the proportion of men engaging in UAI with a partner of unknown HIV status and increases in concordant UAI among either men reporting being diagnosed with HIV or those reporting their last test was HIV negative. This was not seen when we compared our two internet-based samples. However, we did observe a lower rate of reported UAI with partners of unknown HIV status among men not tested HIV positive in 2008 than in 2001 and a rise in reported UAI among men tested positive. Literature also suggests that among MSM in England, there may be a move towards strategic positioning among men diagnosed with HIV. We also found little evidence for this in our data. There were no modality-specific differences in reported UAI among men who had tested for HIV between the two samples. We were however surprised to note a lower reported number of sexual partner numbers among men never having tested and those whose last HIV test was negative (but not those diagnosed HIV positive) in the 2008 sample compared to the 2001 group and a significant increase in any UAI specifically among men with diagnosed HIV.

### Limitations

First, this research involved the analysis of data from two convenience samples of men. This is also the case with other behavioural surveys of MSM which is inevitable given the lack of a sampling frame for this population. However, our samples were very similar in terms of region of residence and ethnicity to the demographic profile of MSM within the National Survey of Sexual Attitudes and Lifestyles—a probability sample survey of British adults conducted in 1999–2001 (Evans et al. 2007). In both years, our sample was however slightly older and better educated than the national sample.

Second, our analysis of differences between the two samples is highly constrained because different sites were used for recruitment in the 2 years. Compared with the 2001 sample, the 2008 sample was much smaller and contained more London residents and was older, more ethnically diverse and better educated. This may reflect differences in the user profiles of the web sites, changes in the uptake of the internet over this decade (Wilkinson and Thelwall 2010) and/or real changes in the demographic profile of MSM in England. For example, the increased proportion of men living with HIV may reflect an increase in the use of the internet to find sexual partners by men diagnosed with HIV. Although we adjusted for the potentially confounding effects of residence, age, ethnic group and education level, it is possible that residual confounding remains which may explain all or part of the observed changes and obscure other real but undetected changes. For example, our 2001 survey (recruited on Gay.com) may have recruited from a more or less at-risk segment of the population than our 2008 survey (recruited on Gaydar and Pink News), a change which may counteract real population level behavioural changes in the other direction. Many of these sampling problems are not specific to web-based surveys. Year-on-year comparisons derived from surveys conducted in gay venues or in gyms will also be affected by establishments opening and closing and changes in the profile of users.

Third, our data relied on self-reports. Cultures of risk disclosure may have altered between 2001 and 2008. This may have been a result of increasing familiarity with community surveying or with secular changes in gay safer sex cultures. There may also have been some misreporting of HIV status as well as some sero-conversions following test results. We did not collect data on time since last HIV test and so could not determine whether HIV tests preceded or followed sexual risk behaviours, so some of the allocations to sero-concordant or discordant behaviours will be invalid. This reduces the power of the study to observe change but is unlikely to introduce other forms of bias. However, the overall lack of observed change in aggregate behaviour aligns well with other surveys of MSM in England (Knussen et al. 2011; Lattimore et al. 2011). We did not collect data on whether the behaviours reported on occurred within regular or casual relationships and so cannot examine for example whether an HIV-negative man reporting UAI with a partner thought to be HIV-negative men represents negotiated safety as it is commonly understood (Kippax et al. 1993).

Finally, it may be that HIV testing uptake between 2001 and 2008 was disproportionately among men at greater risk of HIV, which would include men more likely to engage in anal intercourse. This would result in fewer men in the remaining never tested group who engage in anal intercourse, which accounts for the observed decline in IAI in this group.

### Implications for Research and Policy

Our findings suggest a number of recommendations for research and policy. If confirmed in future research, our research may be indicative of a growing concentration of risk among men with diagnosed HIV, as the HIV-positive population increases. A general trend towards fewer partners and reduced UAI with partners of unknown status may be being accompanied by a trend among men diagnosed with HIV towards more partners and increasing UAI. Such a finding would confirm the ongoing need for prevention programmes to prioritise men with diagnosed HIV and for interventions to be tailored and targeted towards them. At both cross sections, men with diagnosed HIV were more likely to have higher numbers of sexual partners than men not tested HIV positive and more likely to have UAI with partners of unknown HIV status (both receptive and insertive).

Although access to ARVs is universal among MSM with diagnosed HIV in England, viral suppression is not. Among those attending a clinic in Brighton between 2000 and 2006, almost half of all men’s time was spent with a detectable plasma viral load (Fisher et al. 2010). Even if plasma viral load is undetectable, there can be a spike in seminal or anal mucus viral load if another STI is acquired at that site (Sadiq et al. 2005), and among MSM with HIV in Brighton, those diagnosed with another STI were much more likely to have passed on their infection than those without an STI diagnosis (rate ratio 5.3 (95 % CI 2.51–11.29) (Fisher et al. 2010). So men with undetectable (plasma) viral load may be able to pass on HIV if they acquire another STI and are then involved in sexual exposure.

Diagnoses of sexually transmitted infections have increased among homosexually active men in England (as elsewhere in the world) in the last 10 years and are disproportionately high among men with HIV (Health Protection Agency. Sexually transmitted infections in England 2011). As among MSM without HIV, the rate of sexual partner change among MSM with HIV far outstrips the rate of STI screening. Most sexually active MSM are at risk of picking up and passing on STIs. MSM with diagnosed HIV contribute to population HIV risk both directly (through behaviours with a risk of HIV transmission to HIV uninfected men, especially in the presence of another STI) and indirectly (through behaviours that risk the transfer of STIs from one partner to another).

Sexual health interventions with MSM with diagnosed HIV should aim to meet unmet prevention needs based on a needs assessment of the specific population to be targeted or worked with. Unmet sexual health needs are common among men with diagnosed HIV and vary by age, time since diagnosis and treatment stage (Bourne et al. 2012). There is however a clear educational need around the challenge of transferring the Swiss statement about HIV infectivity among STI-free monogamous heterosexual couples (Vernazza et al. 2008) to sexual networks of MSM where open relationships are normative and STIs are common.

At the population level, programmes concerned with MSM with diagnosed HIV should aim (as with MSM without HIV) to increase STI screening, reduce sexual partner change and overlapping partners (particularly unsatisfying partners), reduce the proportion of sexual sessions that feature unwanted anal intercourse and increase condom use in casual anal intercourse and regular partnerships with a risk of HIV transmission. Movements in these directions are unlikely to be achieved without a combination of multi-sectorial education and health public policy making.

Our behavioural findings are broadly in line with other research in this area. Our observation of no overall increase in UAI is consistent with Knussen et al. (2011), observing no change in sexual risk behaviours in Scotland 2002 and 2008, and Lattimore at al., observing no statistically significant change in UAI in London between 1998 and 2008. Among HIV-positive men, we saw an overall increase in the proportion having UAI (from 73.7 to 82.1 %), but no statistically significant change in the proportions having insertive unprotected anal intercourse (IUAI) (62.4 and 59.2 %) or receptive unprotected anal intercourse (RUAI) (66.1 and 68.8 %) specifically. Therefore, of the men who had UAI, the proportion who engaged in both IUAI and RUAI was 74.4 % in 2001 but only 55.9 % in 2008. This suggests an increasing role separation during UAI among positive men as more men had IUAI only (7.6 and 13.3 % of those having UAI in the 2 years) or RUAI only (11.3 and 22.9 %) and fewer had both. However, we did not see significant changes in UAI with partners of specific statuses that would suggest large-scale adoption of either sero-sorting (declining negative and unknown partners for UAI and/or increasing positive partners) or strategic positioning (declining negative and unknown partners specifically for IUAI, but not RUAI and/or the reverse with positive partners).

One explanation for the lack of large overall aggregate changes might be in terms of a trade-off between two countervailing trends. It is possible that a trend towards more UAI among positive men might be offset by countervailing secular trends towards less risk, which may reflect the success of HIV prevention or wider structural changes to the societal position of gay and bisexual men. This is supported by evidence of a trend towards fewer partners. Another explanation is simply that most MSM are much less engaged than we might imagine in debates about HIV risk and managing this via negotiated safety, sero-sorting and strategic positioning so that only a small group of men are influenced by these debates.

Further research is needed to examine the small but increasing proportion of men who do not know their HIV status who engage in insertive UAI with men known to be HIV positive and to examine whether this reflects strategic positioning. There is a continuing need for campaigns to promote HIV testing since, despite an increase in test uptake, almost a third of our participants in 2008 were untested. Our finding of reductions in partner numbers might suggest this is an area for interventions to consolidate existing positive trends and further support HIV-positive men in making decisions to reduce their numbers of sexual partners. Given the lack of evidence reported here for increased take up of negotiated safety, sero-sorting and strategic positioning among MSM, HIV health promotion could offer more support for men for whom these strategies might form an important element in risk reduction. A ‘one-size-fits-all’ approach advocating universal partner reduction and 100 % condom use is unlikely to be effective in achieving substantial population-wide reductions. However, given the possible suggestion that these alternative approaches may not be as well known to men as we might have imagined, interventions must ensure that men understand how these tactics work and the relative risks associated with each strategy. Since the majority of UAI among untested and tested negative men is with men they believe to be HIV negative or whose status they do not know, continuing awareness raising about undiagnosed HIV infection should remain a priority.
